# Anophthalmia, hearing loss, abnormal pituitary development and response to growth hormone therapy in three children with microdeletions of 14q22q23

**DOI:** 10.1186/1755-8166-7-17

**Published:** 2014-02-28

**Authors:** Sophie Brisset, Zuzana Slamova, Petra Dusatkova, Audrey Briand-Suleau, Karen Milcent, Corinne Metay, Martina Simandlova, Zdenek Sumnik, Lucie Tosca, Michel Goossens, Philippe Labrune, Elsa Zemankova, Jan Lebl, Gerard Tachdjian, Zdenek Sedlacek

**Affiliations:** 1 AP-HP, Service d’Histologie Embryologie et Cytogénétique, Hôpital Antoine Béclère, Clamart, France; 2 Department of Biology and Medical Genetics, Charles University 2nd Faculty of Medicine and University Hospital Motol, Prague, Czech Republic; 3 Department of Pediatrics, Charles University 2nd Faculty of Medicine and University Hospital Motol, Prague, Czech Republic; 4 AP-HP, Service de Biochimie-Génétique, Plateforme de Génétique Constitutionnelle, Hôpital Henri Mondor, Créteil, France; 5 AP-HP, Service de Pédiatrie, Hôpital Antoine Béclère, Clamart, France; 6 Genetic and Pediatric Ambulance, Benesov, Czech Republic; 7 INSERM U935, Villejuif, France; 8 Université Paris-Sud, Faculté de Médecine Paris-Sud, Le Kremlin Bicêtre, France; 9 INSERM U955, Université Paris 12, Créteil, France

**Keywords:** Anophthalmia, 14q22q23 microdeletion, *OTX2*, Hearing loss, Pituitary, Growth hormone therapy

## Abstract

**Background:**

Microdeletions of 14q22q23 have been associated with eye abnormalities and pituitary defects. Other phenotypic features in deletion carriers including hearing loss and response to growth hormone therapy are less well recognized. We studied genotype and phenotype of three newly identified children with 14q22q23 deletions, two girls and one boy with bilateral anophthalmia, and compared them with previously published deletion patients and individuals with intragenic defects in genes residing in the region.

**Results:**

The three deletions were *de novo* and ranged in size between 5.8 and 8.9 Mb. All three children lacked one copy of the *OTX2* gene and in one of them the deletion involved also the *BMP4* gene. All three patients presented partial conductive hearing loss which tended to improve with age. Analysis of endocrine and growth phenotypes showed undetectable anterior pituitary, growth hormone deficiency and progressive growth retardation in all three patients. Growth hormone therapy led to partial catch-up growth in two of the three patients but just prevented further height loss in the third.

**Conclusions:**

The pituitary hypoplasia, growth hormone deficiency and growth retardation associated with 14q22q23 microdeletions are very remarkable, and the latter appears to have an atypical response to growth hormone therapy in some of the cases.

## Background

The morphogenesis of midline brain structures, eyes, optic nerves and optic tracts is governed by a cascade of transcription factors including SOX2, OTX2 and BMP4 [1]. Congenital anophthalmia, which is among the most severe consequences of defects in this cascade, is often accompanied by pituitary dysfunction and growth failure due to growth hormone (GH) deficiency [2-6]. Mutations in *SOX2* are the most common cause of anophthalmia, and 10% of their carriers also show growth retardation [1]. The co-occurrence of eye malformations and GH deficiency is the highest (30%) in patients with *OTX2* mutations [3]. *BMP4* defects can induce a similar brain and ocular phenotype [6,7]. Mutations ranging from single nucleotide substitutions to cytogenetically visible deletions have been reported in *OTX2* and *BMP4*, which are both located in 14q22q23. We identified three unrelated patients with anophthalmia, partial hearing loss and pituitary defects due to microdeletions of this region. This gave us a unique opportunity to study not only the ocular phenotypes which are well associated with these rare genetic defects, but also other phenotypic features which are less well characterized.

## Case presentation

### Clinical reports

#### Patient 1

**Figure 1:**
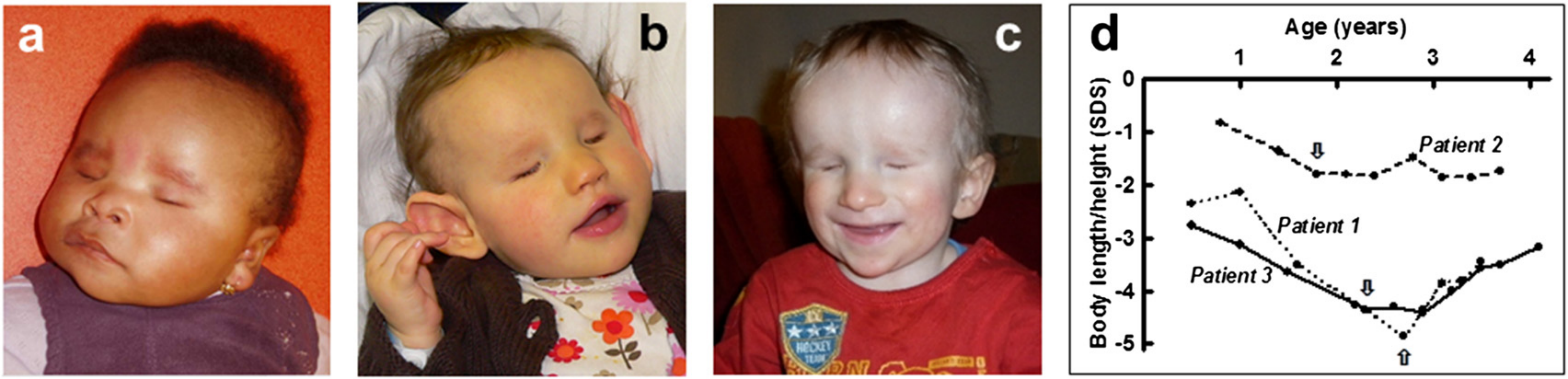
**Facial photographs of the patients and their growth characteristics. (a)** Patient 1 at the age of 6 months; **(b)** Patient 2 at the age of 7 months; **(c)** Patient 3 at the age of 22 months; **(d)** Development of body length with the onset of GH administration indicated by arrows.

The girl (Figure 1a) was born to healthy parents of Mali origin at 38 weeks of gestation by Caesarean section because of abnormal fetal cardiac rhythm. Her birth weight, length and head circumference (HC) were 3,110 g (50th centile), 51 cm (75th centile), and 35.5 cm (75th centile), respectively. She showed bilateral anophthalmia. Brain magnetic resonance imaging (MRI) detected hypoplastic orbits, and absent optic nerves and optic chiasm. The anterior pituitary was undetectable, the posterior pituitary was ectopic with hypoplasia of the pituitary stalk, and the sella was small and flat. She had high forehead, microretrognathia, high arched palate, large ears, persistent hypotonia and right postaxial polydactyly. Cardiac examination revealed a systolic murmur and a perimembranous interventricular septal defect of 3 mm. Skeletal radiography and external genitalia were normal.

After birth, provoked otoacustic emissions were abnormal on the left side. Auditory evoked potentials at 3 years of age found a 60 dB threshold hearing in both ears. Because of persistent middle ear effusion, tympanostomic tubes were inserted, but had to be removed due to chronic otorrhea.A very low serum IGF-I (9 μg/l) and cortisol (<10 nmol/l) levels at 10 days of life suggested GH deficiency and a cortisol function defect (confirmed by ACTH stimulation test). The levels of other pituitary hormones were normal (TSH 3.48 mUI/l, T4 17.8 pmol/l). The growth of Patient 1 progressively deteriorated from −2.4 SD (60 cm) at 6 months to −3.5 SD (71 cm) at 1.6 years and to −4.9 SD (75.5 cm) at 2.7 years of age (Figure 1d). GH therapy at a dose of 35 μg/kg/day initiated at the age of 2.7 years led to an improved growth rate and stepwise normalization of circulating IGF-I (4, 11 and 135 μg/l at the onset and after 3 and 12 months of therapy, respectively; normal age-specific serum IGF-I range is 51–218 μg/l).

#### Patient 2

The girl (Figure 1b) was born to healthy Czech parents in the 41st week of gestation with a weight of 3,500 g (50th centile), length of 52 cm (75th centile), and HC of 33 cm (10th centile). Bilateral anophthalmia and relative microcephaly were noted at birth. Brain MRI revealed absence of optic nerves, optic chiasm and optic tracts. The sella was flat, the pituitary stalk and posterior pituitary were present and normally located, but the anterior pituitary was undetectable. The patient showed profound hypotonia and very large, low-set dysplastic ears, high prominent forehead, high frontal hairline, and wide nose with horizontal nostrils, but no cardiac or genital defects. Radiography revealed the presence of 13 pairs of ribs and unpent arcs of vertebral corpus Th1.

Investigation of otoacustic emissions was not successful in the newborn. At 6 months of age she had normal hearing at the left side and moderate conductive hearing loss at the right side. Stenotic Eustachian tubes likely led to decreased pressure in the middle ear cavity. The hearing loss tended to improve with age.Initially, the growth of the patient was normal. However, it started to decelerate to −0.8 SD (70 cm) at 10 months, −1.4 SD (76.3 cm) at 1.4 years and −1.8 SD (79 cm) at 1.8 years of age (Figure 1d). Endocrine assessment revealed GH deficiency (3.53 ug/l following insulin-induced hypoglycemia at 17 months of age) and IGF-I deficiency (11 ug/l; −1.79 SD) but normal other pituitary functions (TSH 1.19 mIU/l, fT4 12.2 pmol/l, FSH 8.9 IU/l, LH 0.9 IU/l, cortisol 555 nmol/l, prolactin 4.7 ug/l). GH therapy at a dose of 25 μg/kg/day was initiated at the age of 1.8 years. It improved the growth rate and the serum IGF-I level (5, 75 and 116 μg/l at the onset, 12 and 24 months of therapy, respectively) but did not lead to catch-up growth (99 cm at 4 years, i.e. -1.5 SD, Figure 1d).

#### Patient 3

The boy (Figure 1c) was born to healthy Czech parents in the 37th week of gestation by Caesarean section due to intrauterine growth retardation with a weight of 2,060 g and length of 44 cm (both below the 3rd centile for the gestational age), bilateral anophthalmia and marked hypotonia. Brain MRI revealed absent optic chiasm; however, extraocular muscles were preserved. Similarly to Patient 2, the sella was flat, the pituitary stalk and posterior pituitary were present and normally located, but the anterior pituitary was undetectable. Patient 3 showed mesocephaly with prominent narrow forehead, a small narrow face and a wide nasal bridge. He had no apparent morphological ear abnormalities and no cardiac, spinal, abdominal, or genital defects. His bilateral testicular retention required surgical management.

In the neonatal period, the investigation of otoacustic emissions was unsuccessful. At the age of 3 months he showed mild hearing loss of the right ear and medium hearing loss of the left ear. Despite the tympanometry was normal, the hearing loss was apparently conductive due to stenotic ear canals. Also in this patient hearing gradually improved with age.Intrauterine growth retardation was followed by severe postnatal growth failure: at the age of 7 weeks the boy had a length of 48.4 cm (−3.7 SD), and weight of 3,010 g, and his growth had further deteriorated (Figure 1d). He suffered from GH deficiency (1.79 ug/l following clonidine stimulation at 2.3 years of age) and IGF-I deficiency (2 ug/l). Other pituitary functions were apparently normal (TSH 2.75 mIU/l, fT4 11.8 pmol/l, FSH 0.48 IU/l, LH 0.07 IU/l, cortisol 201 nmol/l, prolactin 5.7 ug/l); however, the bilateral testicular retention might be suggestive of a gonadotropin deficiency. GH therapy at a dose of 25 μg/kg/day was initiated at the age of 2.3 years. His height velocity on therapy was atypical, with only a moderate increase within the first year of GH administration but a marked increase thereafter (92.0 cm at 4.1 years, i. e. -3.17 SD, Figure 1d). Serum IGF-I levels were gradually increasing to 6, 19, 33, 56 and 67 μg/l at the onset and during the first two years of therapy.

### Laboratory methods

The study was approved by the local ethics committees and all analyses were performed after proper informed consent. Karyotyping of the patients was performed using standard methods. Array comparative genomic hybridization (CGH) used 180K and 105K CGH arrays (Agilent Technologies) in Patient 1 and Patients 2/3, respectively. The deletions were confirmed in the patients and tested in the parents using fluorescence in situ hybridization (FISH) with probes RP11-533L7/RP11-550 M19 and RP11-550M19 (BlueGnome) in Patients 1 and 2, respectively. In Patient 3 the deletion was confirmed using a 60K CGH array (Agilent Technologies).

## Results

**Figure 2:**
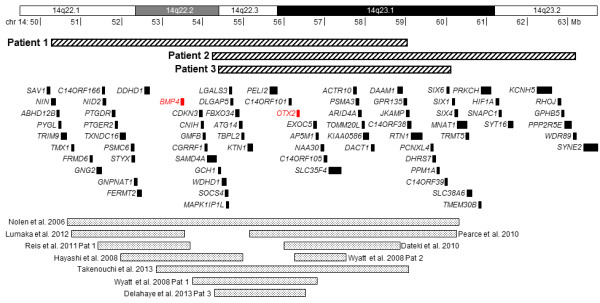
**Schematic representation of the 14q22q23 region affected by deletions in the patients.** The deletions in our patients are shown as thick hatched bars, the deletions overlapping *OTX2* and/or *BMP4* in published cases as dotted bars. Intragenic deletions in *OTX2* or *BMP4* are not shown. Protein-coding genes are indicated by black rectangles (*OTX2* or *BMP4* are in red). The chromosome 14 banding pattern and megabase scale are also included.

Karyotyping did not show chromosome abnormalities in any of the three patients. Array CGH revealed interstitial deletions of 14q22q23 of varying size (Figure 2) in the absence of additional relevant submicroscopic aberrations. Patient 1 carried an 8.8 Mb long deletion (chr14:50293781–59068634, hg18) removing multiple genes including *BMP4* and *OTX2* but not the *SIX* gene cluster. Patient 2 had a deletion of 8.9 Mb (chr14:54251697–63177878) affecting *OTX2* and the *SIX* gene cluster. Patient 3 had a 5.8 Mb long deletion (chr14:54431790–60167626) removing *OTX2* and a part of the *SIX* gene cluster. Using independent methods, all deletions were confirmed in the patients but not in any of the parents, thus indicating the *de novo* nature of the aberrations.

## Discussion

We present a case series of three patients with bilateral anophthalmia caused by microdeletions of 14q22q23. Their phenotype was further characterized by hearing impairment, abnormal pituitary development leading to GH deficiency and early growth failure, and dysmorphic facial features. The overview of phenotypes observed in published cases with 14q22q23 deletions and in our three patients is in Table 1.

**Table 1:** Clinical features in eighteen patients with 14q22q23 deletions

**Report**	**Nolen et al. **[4]	**Hayashi et al. **[11]	**Bakrania et al. **[6]	**Bakrania et al. **[6]	**Wyatt et al. **[8]	**Wyatt et al. **[8]	**Dateki et al. **[3]	**Reis et al. **[7]	**Delahaye et al. **[13]	**Lumaka et al. **[10]	**Lumaka et al. **[10]	**Lumaka et al. **[10]	**Lumaka et al. **[10]	**Pearce et al. **[9]	**Takenouchi et al. **[12]	**Present study**	**Present study**	**Present study**
Patient no.	1	1	1	2	1	2	5	1	3	I-1	II-2	III-5	III-6	1	1	1	2	3
14q deletion	q22.1	q22.1	q22.3	q22.2	q22.2	q22.3	q22.3	q22.1	q22.2	q22.1	q22.1	q22.1	q22.1	q22.3	q22.2	q22.1	q22.3	q22.3
	q23.1	q23.1	q23.2	q23.1	q22.3	q23.1	q23.1	q22.2	q23.1	q22.2	q22.2	q22.2	q22.2	q23.1	q23.1	q23.1	q23.2	q23.1
Sex	M	F	F	M	F	F	M	F	M	M	F	F	F	F	F	F	F	M
Age at last examination	5 yr	18 mo	N.D.	N.D.	19 mo	3 yr	2 yr	6 yr	24 yr	Adult	Adult	13 mo	11 mo	4 mo	3 yr	4 yr	4 yr	4 yr
Anophthamia unilateral (AOU)/bilateral (AOB); microphthalmia unilateral (MOU)/bilateral (MOB)	AOB	-	AOB	AOB	MOB	AOB	AOU/MOU	MOB	MOB	-	-	-	MOB	AOU/MOU	MOB	AOB	AOB	AOB
Optic nerve and/or chiasma and/or optic tracts hypoplasia/agenesis	+	-	+	+	N.D.	N.D.	N.D.	-	N.D.	-	-	-	N.D.	+	-	+	+	+
Cerebral and/or facial midline defects	+	-	+	+	-	-	-	-	+	-	-	+	+	+	-	+	-	-
Pituitary aplasia/hypoplasia	+	-	N.D.	+	-	-	+	-	N.D.	N.D.	N.D.	N.D.	N.D.	-	-	+	+	+
Hormonal deficiencies: growth hormone deficiency (GHD)/hypothyroidism (HT)	GHD	N.D.	HT^#^	-	N.D.	N.D.	GHD	N.D.	N.D.	N.D.	N.D.	-	N.D.	-	-	GHD	GHD	GHD
Prenatal growth	Normal	Normal	N.D.	N.D.	Normal	Normal	Normal	Normal	N.D.	Normal	Normal	Retarded	Retarded	Normal	Normal	Normal	Normal	Retarded
Postnatal growth	Retarded	Retarded	N.D.	N. D.	N.D.	N.D.	Retarded	Retarded	N.D.	Normal	Retarded	Retarded	Retarded	N.A.*	Normal	Retarded	Retarded	Retarded
Microcephaly	+	-	-	+	-	-	+	-	+	-	-	-	+	+	-	-	+	-
Hearing loss/ear anomalies	+	-	-	+	-	-	N.D.	-	-	-	-	+	-	+	-	+	+	+
Undescended testes	+	N.A.	N.A.	+	N.A.	N.A.	N.D.	N.A.	-	-	N.A.	N.A.	N.A.	N.A.	N.A.	N.A.	N.A.	+
Developmental delay/intellectual disability	+	+	+	+	-	+	+	+	+	-	-	+	+	N.A.*	+	+	+	+
Polydactyly/syndactyly	+	+	-	-	-	-	-	-	-	+	+	-	+	-	-	+	-	-
Major additional extracranial symptoms								SHORT syndrome; partial lipodystrophy	Renal			-		Duodenal atresia	Profound hypotonia			

N.D., not described; N.A., not applicable; *patient too young; ^#^not specified if secondary (central) or primary (peripheral).

Similarly to Figure 2, this table does not list patients with deletions assessed using karyotyping in whom the genes affected are uncertain and small deletions affecting only *OTX2* or *BMP4* exons. The deletions in patients described by Bakrania et al., 2008 were identified using karyotyping and MLPA analysis was used to show that both deletions affected both *OTX2* and *BMP4*.

The 14q22q23 region is critical for eye and pituitary development. Anophthalmia and other ocular anomalies were associated with heterozygous defects in *OTX2*[3,8,9] or *BMP4*[7,10,11], and also with deletions involving both these genes [4,6,12]. While *OTX2* was deleted in all our patients, *BMP4* was deleted only in Patient 1 (Figure 2). Nevertheless, the ocular phenotype was similar in all three children. The phenotypic effect of *OTX2* and *BMP4* disruptions is very variable ranging between anophthalmia/microphthalmia, corneal opacity and no abnormality at all, even in family members with the same mutation [2,5,9-13], and the phenotype does not have to be more severe in patients with combined *OTX2*/*BMP4* defects [4,12]. Patients 2 and 3 also lacked *SIX6*; however, defects of this candidate gene have not been identified in anophthalmia [1].

All three patients suffered from transient partial conductive hearing loss. Previous reports of patients with 14q22q23 deletions were inconsistent, varying from not mentioning the hearing status over normal function [7,12] to severe unilateral hearing loss [4,9], indicating very variable expressivity. We speculate that skeletal abnormalities of the facial-cranial junction of the skull associated with anophthalmia due to the *OTX2* defect can induce stenosis of ear canals and/or Eustachian tubes and hearing impairment. Changing proportions of these structures during growth could also explain the gradual improvement of hearing with age observed in our patients. Interestingly, Patient 2 with large low-set dysplastic ears was the only our patient with *SIX1* disruption. Homozygous knockout of this gene in mice causes malformations of the auditory system including outer ears [14]. Deletions in two published patients also involved *SIX1* and were associated with malformed ears, although differently from Patient 2 [4,9]. *OTX2* defects themselves could also contribute to ear anomalies [15].

Our patients showed abnormal pituitary development, GH deficiency and growth retardation. Normal birth parameters and postnatal growth failure similar to that in Patients 1 and 2 were reported in some cases with 14q22q23 deletions [3,4,11,12]. On the other hand, Patient 3 suffered from severe intrauterine growth retardation without postnatal catch-up. An improvement of growth was evident after GH therapy in all our patients although the response varied. In Patients 1 and 3 the therapy induced an increase of growth velocity and improvement of their height, whereas in Patient 2 it just prevented further growth deterioration. However, in Patient 2 the growth failure was least pronounced with height of −1.8 SD at the start of the GH therapy, and this fact could influence the GH response. Similarly, two published 14q22q23 deletion patients treated with GH remained with their height at −2 SD after five and three years of therapy [4,13], and the height of a boy with a missense *OTX2* mutation remained at −2.3 SD after eight years of therapy [2]. As no reports are currently available on final height of 14q22q23 deletion patients, it remains to be seen if Patients 1 and 3 correct their growth failure completely or if they just reach the current height range of Patient 2. Differences in the deleted genes do not offer an obvious explanation for the differences in responsiveness to the GH therapy. Two genes involved in pituitary development, *OTX2* and *SIX6*, were disrupted in Patients 2 and 3. Patient 1 had a deletion of *OTX2* and *BMP4*, but not of *SIX6*. The differences in pituitary morphology between Patient 1 and Patients 2 and 3 can also be attributed solely to the variability seen among carriers of isolated *OTX2* defects [1,3].

Finally, polydactyly was present in Patient 1 with a deletion of *BMP4*. This gene plays an important role in the onset of endochondral bone formation in humans, and its mutations were associated with polysyndactyly [4,11].

Recently, two papers were published describing patients with deletions overlapping the proximal and distal part of the 14q22q23 region [16,17]. A family with Frias syndrome carried a deletion of 14q22.1q22.3 spanning the interval between *GNG2* and *KTN1*, with *BMP4* haploinsufficiency being likely responsible for the phenotype which included hypoplasia of corpus callosum, minor ocular anomalies, specific tooth defects, digit anomalies, short stature and intellectual impairment [16]. The other deletion involving 14q22.3q23.2 and extending from *PSMA3* over the *SIX* cluster to *SYNE2* was identified in a patient with facial dysmorphism, choanal atresia, esophageal reflux, defects of hands and feet, seizures and intellectual disability [17]. Interestingly, in the latter case neither microphthalmia/anophthalmia nor pituitary anomalies were present (MRI was normal), and neither *OTX2* nor *BMP4* were deleted. These papers further illustrate the intra- and interfamilial variability which, together with biased and incomplete reporting of the phenotypes, complicates the genotype-phenotype correlation in patients with unique deletions affecting multiple genes which participate in different molecular pathways [16,17].

## Conclusions

Our case series study of three patients with deletions of 14q22q23 demonstrated the phenotypic features and variable expressivity of this genetic defect. Comparison with previously published patients with similar microdeletions and mutations in the *OTX2* gene suggests that most symptoms presented by affected patients could be attributed to *OTX2* haploinsufficiency. Growth retardation due to GH deficiency is very remarkable in these patients and GH treatment can increase the growth velocity especially in the most severe cases but the response can be atypical. The hearing impairment can be transient and improves with age.

## Consent

Written informed consent was obtained from the parents of the patients for publication of this report and the accompanying images. A copy of the written consent is available for review by the Editor-in-Chief of this journal.

## Competing interests

The authors declare that they have no competing interests.

## Authors’ contributions

SB, ZSl, PD, ABS, CM, LT and MG carried out the cytogenetic and molecular cytogenetic studies; JL, ZSu, MS, EZ, SB, CM, KM and PL participated in the analysis of the phenotype and genetic counseling; PD, ZSe, JL and SB drafted the manuscript; ZSu edited the manuscript; ZSe, JL and GT conceived, designed and coordinated the study. All authors read and approved the final manuscript.
